# Managing infectious diarrhea among young children in community pharmacies in Saudi Arabia and the implications for AMR

**DOI:** 10.3389/fped.2024.1342493

**Published:** 2024-03-18

**Authors:** Faris S. Alnezary, Amira R. Alamri, Rafa D. Alrehaili, Dina S. Alnizari, Fahad Alzahrani, Mansour Mahmoud, Masaad S. Almutairi, Amanj Kurdi, Brian Godman

**Affiliations:** ^1^ Department of Pharmacy Practice, College of Pharmacy, Taibah University, Madinah, Saudi Arabia; ^2^ Department of Pharmacy Practice, College of Pharmacy, Qassim University, Qassim, Saudi Arabia; ^3^ Strathclyde Institute of Pharmacy and Biomedical Sciences, University of Strathclyde, Glasgow, United Kingdom; ^4^ Department of Public Health Pharmacy and Management, School of Pharmacy, Sefako Makgatho Health Sciences University, Pretoria, South Africa; ^5^ Department of Clinical Pharmacy, College of Pharmacy, Hawler Medical University, Erbil, Iraq; ^6^ Department of Clinical Pharmacy, College of Pharmacy, Al-Kitab University, Kirkuk, Iraq

**Keywords:** antibiotics, appropriate care, antimicrobial resistance, pediatric diarrhea, community pharmacy, public health, Saudi Arabia

## Abstract

**Introduction:**

Diarrhea remains a major global health issue for children under five, contributing substantially to morbidity and mortality. Community pharmacists play a pivotal role in the management of these children; however, their competence in managing childhood diarrhea in Saudi Arabia is under-researched. This is important to ensure optimal patient care.

**Method:**

Simulated patients (SPs) presenting with three pediatric diarrhea scenarios were used to evaluate pharmacists’ practice in terms of their counselling, history taking, over-the-counter (OTC) prescribing, medication instructions, diet/fluid advice, and/or information provision. Pharmacists’ practice was categorized into adequate, less adequate, and poor.

**Results:**

182 community pharmacists, primarily male and non-Saudi, participated in the study, of which 60% were in chain pharmacies. Only 5% showed adequate practice in currently managing pediatric diarrhea. Of the 182 simulated patient visits, 62% received medication in all three scenarios and 20% were referred to physicians, with 16% of pharmacists failing to provide any form of intervention. The main medications recommended were kaolin (34%), pectin (34%) and metronidazole (11%). While most pharmacists (86%) asked about the patient's identity and age, 15% provided incorrect management information, 16% failed to provide guidance on the prescribed medicines, and 18% dispensed antimicrobials without a valid prescription.

**Conclusion:**

A high level of inadequate management of pediatric diarrhea in Saudi Arabia was observed. This highlights the need for extensive training to improve community pharmacists’ practice in service delivery including providing counselling and advice on the appropriate management of childhood diarrhea. The latter is particularly important to reduce antimicrobial resistance.

## 1 Introduction

Globally, pediatric diarrhea accounts for more than 1.7 billion cases annually and is the second leading cause of mortality in children aged five and below (1). In 2017, pediatric diarrhea was responsible for an estimated 533,768 deaths globally in children under five years of age, giving an estimated mortality rate of 78.4 (70.1–87.1) per 100,000 children (2). Moreover, dehydration caused by diarrhea contributes significantly to approximately 1.5–2.5 million deaths annually among young children (1). Diarrhea is characterized by an increased frequency of defecation, with the stools being excessively soft or watery. The World Health Organization categorizes diarrhea into three clinical types, with acute watery diarrhea being the most common. This type is transient, lasting several hours to a few days, and primarily causes dehydration, along with potential symptoms including nausea, vomiting, fever, weight loss, pale complexion, malaise, and abdominal discomfort (1, 3).

Infectious agents such as viruses, bacteria, and protozoa, often encountered during travel or through contaminated food, are the primary cause of diarrhea. Sudden onset characterizes infection-induced diarrhea, with some antibiotic-associated infections, such as *Clostridioides difficile*, being potential causes (4, 5). Management strategies aim to identify the root cause of the diarrhea, initiate timely treatment and prevent severe dehydration, malnutrition, and death (6, 7).

Treatment primarily focuses on restoring electrolyte and fluid balance (1, 5). Antidiarrheal medications have proven effective in certain cases among adult patients and older children. Therapeutic options include oral rehydration solution (ORS) and/or zinc supplementation, as well as kaolin, loperamide, and probiotics (1, 8, 9). ORS and/or zinc supplements are widely used to manage dehydration and restore electrolyte balance, reducing the frequency of diarrhea episodes. Kaolin, often used with ORS, aids in water absorption, toxin, and germ removal in the gastrointestinal system. The U.S. Food and Drug Administration advises against using loperamide, an antimotility agent, in infants and children under two because of potential harm (10). Probiotics may also prevent antibiotic-induced diarrhea (11). Severe cases may require referral to a physician based on specific criteria, although mild cases often respond to ORS and/or zinc supplementation (1, 3). Nutrient-rich foods and adequate hydration are also beneficial (1).

Antibiotics are generally not recommended as a first-line treatment for infectious diarrhea, unless there is a specific bacterial infection causing the symptoms (12, 13). A recent study reported that rehydration is the critical treatment for children with acute diarrhea as symptoms usually resolve without antibiotics (14). However, when empirically administered especially if symptoms persist in children with acute bloody diarrhea, studies have found that antibiotic treatment can manage clinical symptoms and reduce the duration of diarrheal illness (12, 15).

Patients with diarrhea who are neglected, or receive insufficient treatment, may encounter complications including increasing morbidity, which negatively impacts on their quality of life. Community pharmacists can intervene effectively at this stage due to their increasing role in preventing and diagnosing childhood diarrhea, providing education and referral services (16). As accessible healthcare providers, they are pivotal in community health, endorsed during the recent COVID-19 pandemic (17, 18). However, there is a currently a lack of knowledge regarding community pharmacists’ management of acute childhood diarrhea in Saudi Arabia. It remains uncertain whether pharmacists are delivering appropriate management or facing challenges in this area. Identified common obstacles include insufficient patient history acquisition, inappropriate medication suggestions including antibiotics, suboptimal consultation practices, and limited public understanding of community pharmacists’ roles (16, 19). There have also been concerns with the dispensing of antibiotics without a prescription without a thorough history, rehydration and other approaches first as well as a referral to a physician if needed. The dispensing of antibiotics without a prescription has reduced appreciably in Saudi Arabia in recent years following the tightening of the regulations, greater monitoring of community pharmacy activities as well as potentially considerable financial penalties for abusing the law forbidding such practices (20). However, we are aware that inappropriate practices can return if over time there is less monitoring of community pharmacy activities coupled with continued pressure from patients to dispense antibiotics without a prescription (21–23). Such pressures were heightened during the recent COVID-19 pandemic in Saudi Arabia (24).

In view of ongoing controversies including the dispensing of antibiotics without a prescription, as well as concerns with the current management of children with diarrhea in this high priority area, we sought to ascertain current advice and management among community pharmacists in Saudi Arabia for the treatment of these children. This is particularly important due to lack of guidelines on this topic in Saudi Arabia coupled with high morbidity and mortality. Consequently, the objective of this study was to assess the practice, proficiency, and willingness of community pharmacists in Saudi Arabia in acquiring essential information and implementing appropriate interventions for managing childhood diarrhea. This includes promoting optimal management including rehydration and not dispensing any antibiotics without a referral and prescription from a physician, building on the earlier study of Merghani Ali et al. (16). Reducing inappropriate dispensing of antibiotics in ambulatory care will reduce antibiotic resistance (AMR) (12, 22).

## 2 Materials and methods

### 2.1 Study design

This study employed a cross-sectional, observational design utilizing a simulated patient (SP) methodology, which is a recognized tool for evaluating community pharmacists’ (CPs) skills and practices in real-world settings (25). Specifically, we employed a covert simulated patient approach, a novel method in observational research. Using simulated patients allows the observation of practitioners’ actual responses to predetermined scenarios (26). Consequently, is seen as a more reliable method to ascertain current practices than self-administered questionnaires (27). The SPs assessed pharmacists’ practice and skills, as well as the quality of their counselling in real-life encounters using predetermined case scenarios, which is similar to the approach of Merghani et al. (16).

The study targeted a convenience sample of community pharmacies in Madinah Province, including both chain and independent pharmacies. This province was chosen for this study as it has a diverse population consisting of people from many countries who come to visit the Prophet's Mosque. In addition, to the best of our knowledge, current practices among community pharmacists towards childhood diarrhea in the province has not been studied before. No specific type of pharmacy was excluded from the study.

### 2.2 Sampling method and size

The study included all the districts of Madinah, utilizing a simple random selection methodology. The sample size was calculated to be 165 using the Raosoft sample size calculator, based on a 95% confidence interval, a 5% margin of error, an estimated population size of around 288 community pharmacies, and a response distribution of 50% (28). To enhance the statistical significance of the findings, an additional 17 pharmacies were included, resulting in a total sample size of 182. This increased the total number of pharmacists approached allowing for greater confidence and representativeness.

The sampling frame comprised all eligible and accessible community pharmacies in our region. To avoid duplication, any pharmacy branches or outlets belonging to the same parent company were excluded, with only the primary location selected. Each pharmacy could only be visited once across the three case scenarios to prevent re-surveying the same pharmacist.

During the visits, the simulated patients asked for “the pharmacist on duty” without specifying individual practitioners. The simulated patient exclusively interacted with the first pharmacist who they became engaged with, even if others later offered help. Only one pharmacist was assessed per pharmacy to maintain independence of the observations. Details were recorded reflecting the interaction with the pharmacist encountered rather than the whole pharmacy staff.

### 2.3 Simulated patients

A team of three pharmacy students from the College of Pharmacy in Taibah University, trained as SPs, visited the community pharmacies in Madinah. The SP acted as a relative for the patient and carried out the simulated scenario. Each participant conducted a total of 60 visits, with 20 visits allocated to each scenario. The overall number of visits, including additional visits, amounted to 182. The SPs were trained to only provide information when specifically asked. The visits began in March 2023 and lasted for over three weeks.

Three hypothetical patient scenarios (Table 1) involving young children diarrhea were developed based on a thorough review of the reference guide “Symptoms in the Pharmacy” (3). The three scenarios were designed to assess different aspects of community pharmacists’ practice in managing childhood diarrhea. This included the ability of community pharmacists to identify patients requiring a physician referral (scenario 1), select appropriate OTC treatments (scenario 2), and provide counseling (scenario 3). Relevant assessment points and questions were incorporated into the scenarios by categorizing information from published literature on key considerations for triaging and advising patients with diarrhea (16, 19).

**Table 1:** The simulated patient scenarios.

	Possible causes	Case	Questions	Additional information
1	The cause is unknown.	• Your 8-month-old son has had watery diarrhea 3–4 times per day since yesterday, approximately 16 h ago. • He also has a fever up to 101°F (38°C), appears pale, and is feeding poorly—taking less than half his usual bottle volume. • No one else in the family is sick. You have not given any medicine for the diarrhea or fever, nor seen a doctor yet. • You go to the pharmacy asking what to do for your son's diarrhea. If he/she asks you about the following, answer the following, and then fill out the given form.	1. Duration: since yesterday 2. Frequency of stool: maybe 3–4 times a day, I don't know exactly 3. Other symptoms: pale skin, and fever 4. Other family members affected: no 5. Changes in the feeding pattern: poor feeding (not accepting feeding as usual) 6. Medicine already tried & visiting the doctor: no	
2	The cause is eating fast food 2 days ago with her older siblings, he got diarrhea the next day and take ORS from the pharmacy but the symptoms didn't relieve.	• Your 5-year-old brother has had watery diarrhea more than 4 times per day for the past 2 days. • He also appears pale and lethargic, with dry lips and mouth. • No one else in the family is sick. • His appetite is reduced—he ate fast food yesterday which may have contributed to the diarrhea. • You tried giving him one glass of oral rehydration solution (ORS) after breakfast yesterday, but his diarrhea has persisted. • You go to the pharmacy asking what to do for your brother's ongoing diarrhea.You will then wait for the pharmacist to respond. If he/ she asks you about the following, answer the following, and then fill out the given form.	1. Duration: 2 days 2. Frequency of stool: >4 times 3. Other symptoms: pale skin, lethargy, weakness, and dry mouth and lips 4. Other family members affected: no 5. Changes in food: yes, eating fast food 6. Medicine already tried: yes, ORS. 7. How did you give him the ORS: put it in a glass of water and drank it once after breakfast.	
3	Got diarrhea after his friend at daycare had it.	• Your 3-year-old brother has had watery diarrhea more than twice per day since last night, approximately 18 h ago, after returning from daycare. • He had his first loose stool around 8PM yesterday evening. • In addition to the diarrhea, he seems fatigued and less energetic than usual. • No other family members are affected. • His diet has been normal with no recent food changes. • He has not been given any medication for the diarrhea yet, nor been seen by a doctor. • You go to the pharmacy asking what to do for your brother's ongoing diarrhea. • You will then wait for the pharmacist to respond. • If he/she asks you about the following, answer the following, and then fill out the given form.	1. Duration: since yesterday, at night after he came from daycare 2. Frequency of stool: >2 times 3. Other symptoms: fatigue 4. Other family members affected: no 5. Changes in food: no 6. Medicine already tried & visiting the doctor: no	Onset: started when he came from daycare.

Case 1 involved an 8-month-old infant boy who had been suffering from watery stools up to 4 times per day that started suddenly yesterday. This simulated case is intended to assess whether the pharmacist makes the appropriate referral to a physician, given medical guidelines stating that all pediatric patients under 1 year of age presenting with diarrhea should be evaluated by a doctor.

Case 2 involved a 5-year-old boy with watery stools ongoing for 2 days. His sibling administered oral rehydration solution from the pharmacy, but diarrhea persisted over 4 times daily. The child exhibits concerning symptoms including paleness, lethargy, weakness, and dry mouth. The sibling returns to the pharmacy seeking additional treatment, providing details if probed. The case assesses whether the pharmacist recognizes the need for doctor referral given signs of dehydration.

Case 3 involves a 3-year-old boy with watery stools for 2 days, starting after returning from daycare. He has had over 2 loose bowel movements daily and seems fatigued, indicating likely infectious diarrhea. No medications have been tried yet. His sibling goes to the pharmacy seeking treatment. If probed, the sibling provides details—no dietary changes and no other family members affected. The case assesses the pharmacist's ability to select appropriate therapy for a young child's probable infectious diarrhea.

### 2.4 Data collection form

To minimize potential biases caused by incomplete information, every visit was thoroughly documented and later transcribed onto a designated form. The form consisted of eight sections: (i) pharmacy information [location and type (independent vs. chain pharmacy)], (ii) pharmacist information (nationality and gender), (iii) history taking, (iv) the treatment(s) dispensed, (v) type of action and advice on suggested treatments, (vi) instructions given about any medicine dispensed, (vii) advice about food and fluid intake and (viii) any additional information.

The research team, experienced in developing pharmacy assessment standards, created specific criteria to evaluate pharmacists’ willingness and attitudes toward consumers via simulated patients. The criteria measured active listening skills, nature/quantity of questions, extent of counseling, professional demeanor, and tone of voice. Active listening was assessed through eye contact, verbal acknowledgements, and clarifying questions. Open/closed-ended questions were quantified. Counseling was evaluated by word count. Pharmacists’ demeanor was subjectively rated on a 3-point scale. Their tone of voice was rated on a 5-point scale for audibility, clarity, pace and warmth.

Pharmacists’ level of interest in assisting the simulated patients was categorized as follows (3-point scale):

• High interest: displayed exceptional engagement through open-ended questions, active listening, detailed explanations, patience, and warm tone. In addition, spent considerable time interacting with the patient.
• Typical interest: addressed the patient politely with medium engagement, asked some close-ended questions, provided a straightforward response, and did not elaborate extensively.
• No/limited interest: appeared detached, impatient, or unwilling to substantially assist the patient. Interaction was brief.

Most criteria were evaluated using a binary scale: yes or no. Another author reviewed the recorded information for each visit and the associated data to add robustness to the subjective assessments.

### 2.5 Data collection procedures

Standardized forms were created to enable systematic documentation of relevant metrics during the simulated patient encounters. The forms allowed capture of details on history taking, pharmacist questioning, treatment or referrals advised, medication counseling, and adherence to pediatric diarrhea management guidelines.

Simulated patients underwent practice sessions to standardize their presentation and questioning of pharmacists across visits. Completed forms were subsequently reviewed by the study team for completeness, clarity, and correctness.

To minimize inter-rater variability, the same simulated patient completed all encounters for a given case scenario. Visits were scheduled across different times of day over a 3-month period to account for potential variations in pharmacist availability and to prevent detection.

### 2.6 Outcomes

#### 2.6.1 Primary outcome

The primary outcome of this study was to assess the practice of community pharmacists in acquiring necessary information and executing appropriate interventions for managing pediatric diarrhea. The practice was determined by a total score of 10 for each of the three assessed primary components: patient assessment, intervention, and professional behavior. The scores for each component were subsequently categorized into three categories: Adequate practice (7–10), Less adequate practice (4–6), and poor practice (<4) in line with previous publications (29).

#### 2.6.2 Secondary outcomes

Secondary outcomes included the frequency of antimicrobial dispensing without a prescription, pharmacists’ willingness to assist the patient, comparison of practices between chain and independent pharmacies, and the potential influence of the pharmacy's geographical location on its practice.

Pharmacies were broken down into chains and independent pharmacies as there can be differences in the extent of dispensing of antibiotics without a prescription depending on the nature of the pharmacy (30).

### 2.7 Data analysis

Descriptive statistics were employed to present the data, and categorical variables were reported as frequency and percentages. To assess the relationship between pharmacist's characteristics and practice categories, the Pearson Chi-Square was applied. The significance level was set at *p*-value <0.05. The statistical analysis was performed using IBM SPSS software version 25.

### 2.8 Ethical approval

This study received ethical approval from the Institutional Research Ethics Committee at Taibah University (Ref number: COPTU-REC-55-20230224), covering all aspects of the research methodology. Throughout the investigation, the anonymity of participating community pharmacists was ensured by having the chief investigator (FSA) assign a number to each one, which was kept securely with password protection.

## 3 Results

### 3.1 Demographics and locations

The majority of the participating pharmacists were male (96.2%, *n* = 175). In terms of their nationality, an appreciable portion were non-Saudi (85.2%, *n* = 155). A slightly higher percentage were chain pharmacies (59.9%, *n* = 109) compared to independent ones (40.1%, *n* = 73). Most of the pharmacies were located away from Haram (93.4%, *n* = 170) as opposed to those located close to Haram (6.6%, *n* = 12). Table 2 explains the demographic characteristics of the study population.

**Table 2:** Demographic and other variables.

Variables N (%)	*N* (%)
Gender	
Male	175 (96.2)
Female	7 (3.8)
Nationality	
Saudi	27 (14.8)
Non-Saudi	155 (85.2)
Type of pharmacy	
Independent	73 (40.1)
Chain	109 (59.9)
Pharmacy location	
Close to Haram	170 (93.4)
Away from Haram	12 (6.6)
Period of visit	
Morning (8 a.m.–12 p.m.)	30 (16.5)
Afternoon (12 p.m.–4 p.m.)	39 (21.4)
Evening (4 p.m.–8 p.m.)	51 (28)
Night (8 p.m.–12 a.m.)	62 (34.1)

### 3.2 Community pharmacists’ practice assessment

**Figure 1:**
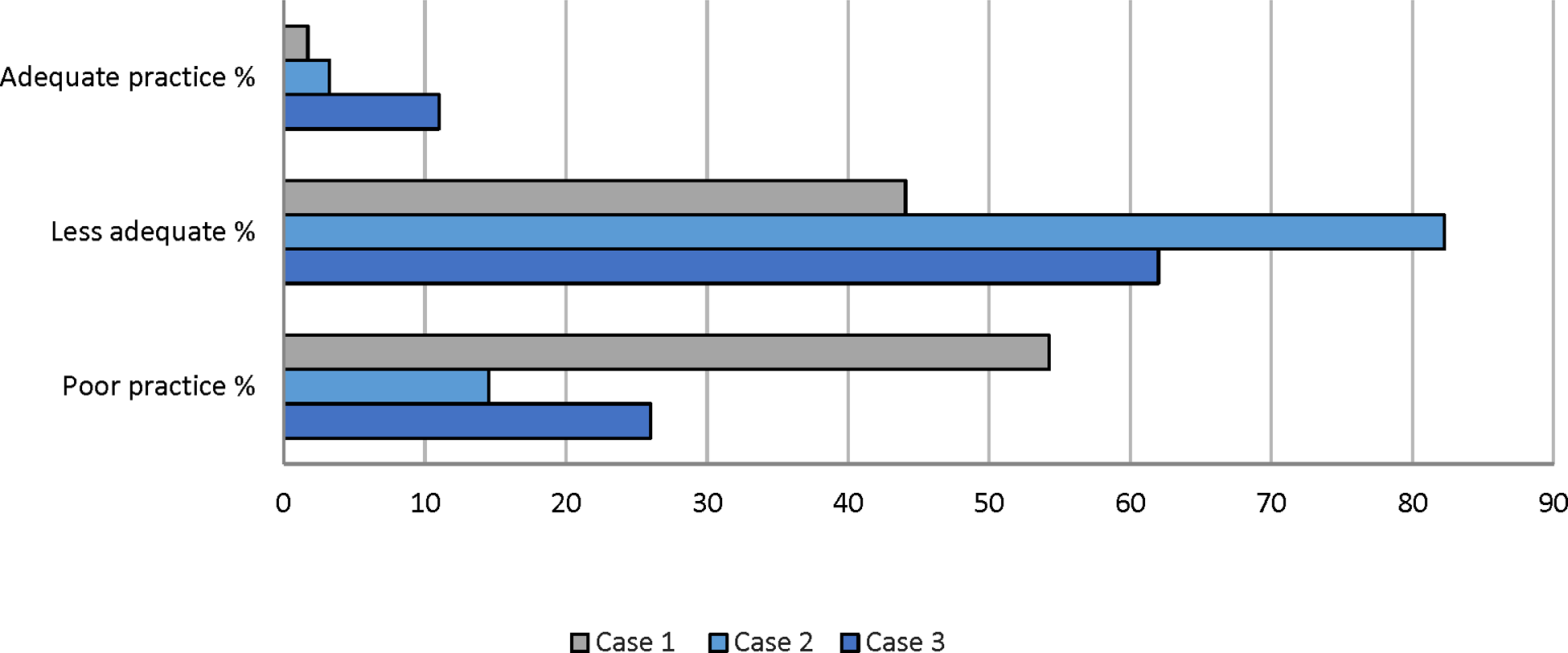
Percentage (%) distribution for assessment of community pharmacists’ practice across all scenarios. Scores were classified into three categories: Adequate practice (7–10), Less adequate practice (4–6), and Poor practice (<4).

Across all three scenarios, the average percentage of pharmacists demonstrating adequate, less adequate, and poor practice were 5% (*n* = 9), 63% (*n* = 115), and 32% (*n* = 58), respectively. The third scenario had the highest rate of adequate practice at 11% (*n* = 7), while the first and second cases had lower rates at 2% (*n* = 1) and 3% (*n* = 2) respectively. The second scenario resulted in a high percentage of less adequate practice (82%, *n* = 51) compared to 44% (*n* = 26) in the first scenario and 62% (*n* = 38) in the third scenario. Regarding poor practice, the first scenario elicited the highest rate at 54% (*n* = 32), while the second had the lowest rate at 15% (*n* = 9). The third scenario exhibited an intermediate level of poor practice at 26% (*n* = 16) (Figure 1). In summary, the simulated cases revealed variability in pharmacists’ referral and treatment decisions based on the three differing pediatric diarrhea scenarios.

### 3.3 Outcomes of simulated patient visits: medication provision, referrals, and advice

**Figure 2:**
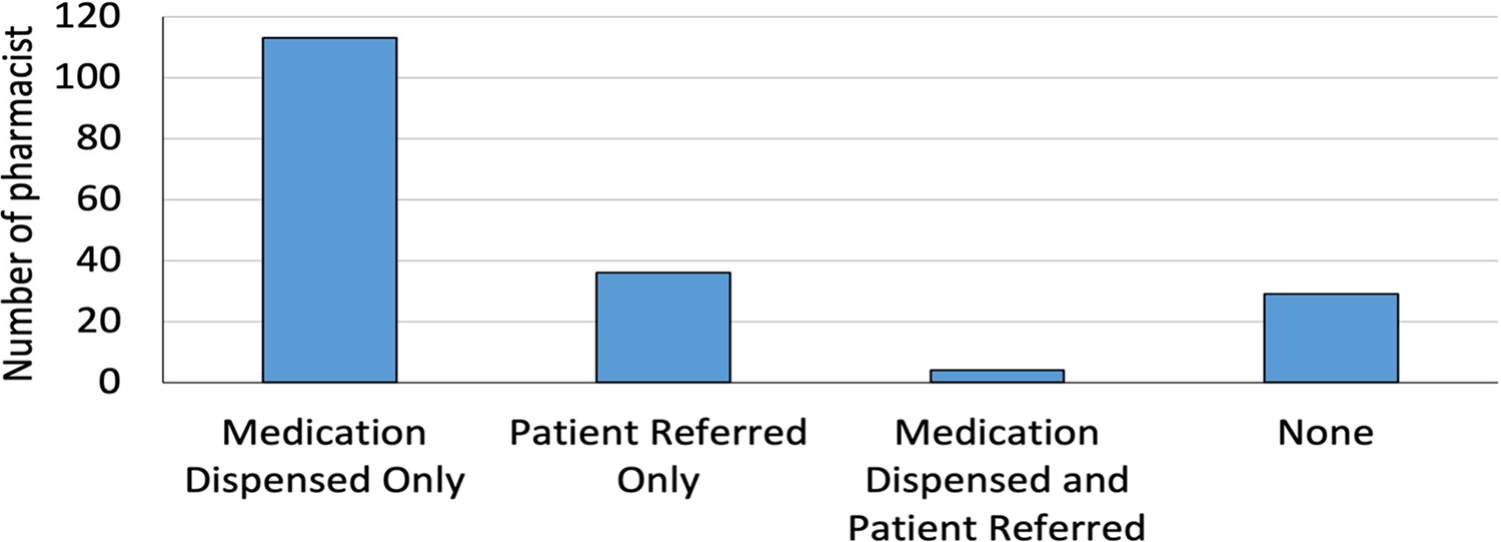
Type of outcome from pharmacy visits in all scenarios.

Of the 182 simulated patient visits, 62% (*n* = 113) received medication in all three scenarios. Only 20% (*n* = 36) of the total cases were referred to physicians for further evaluation. In the total sample of pharmacists, 16% (*n* = 29) failed to provide any form of intervention such as a recommended treatment or referral, and merely stated that they were out of medication. In a small subset of cases (2%, *n* = 4), pharmacists recommended medicines and advised patients to seek medical consultation. Furthermore, in the first and third scenarios, only 37% (*n* = 22) and 8% (*n* = 5) of the community pharmacists, respectively, made appropriate referrals to physicians. In the second scenario, 32 out of 62 pharmacists failed to provide appropriate therapy for diarrhea (Figure 2).

### 3.4 Type of medication recommended

**Figure 3:**
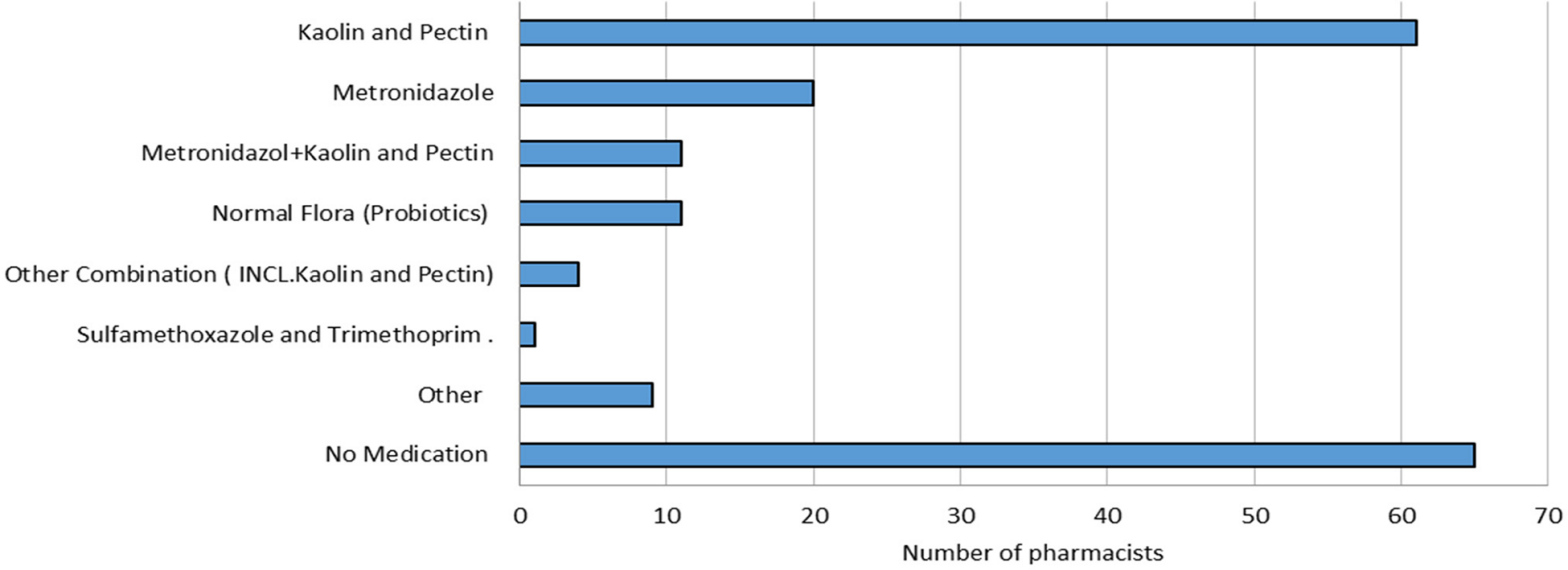
Recommended treatment options for all scenarios.

The main medications recommended were kaolin and pectin (34%, *n* = 61) as well as metronidazole (11%, *n* = 20). In 6% (*n* = 11) of cases, both metronidazole as well as kaolin and pectin were administered along with probiotics. The recommended treatment options among participating pharmacists for all given scenarios are illustrated in Figure 3.

### 3.5 Pharmacists’ practice toward patients’ assessment and treatment

A majority of pharmacists (86%, *n* = 156) asked about the patient's identity, while 90% (*n* = 163) asked about the patient's age. A small number of pharmacists (8%, *n* = 15) provided incorrect information relating to diagnosis or treatment. Of the total sample, 49% (*n* = 91) did not provide guidance on any medicines dispensed, while 42% (*n* = 51) gave appropriate counseling. Regarding the ORS, only 37 (20%) of pharmacists recommended its use, and of these, 24 (13%) provided adequate instructions on ORS usage.

### 3.6 Secondary outcomes

**Figure 4:**
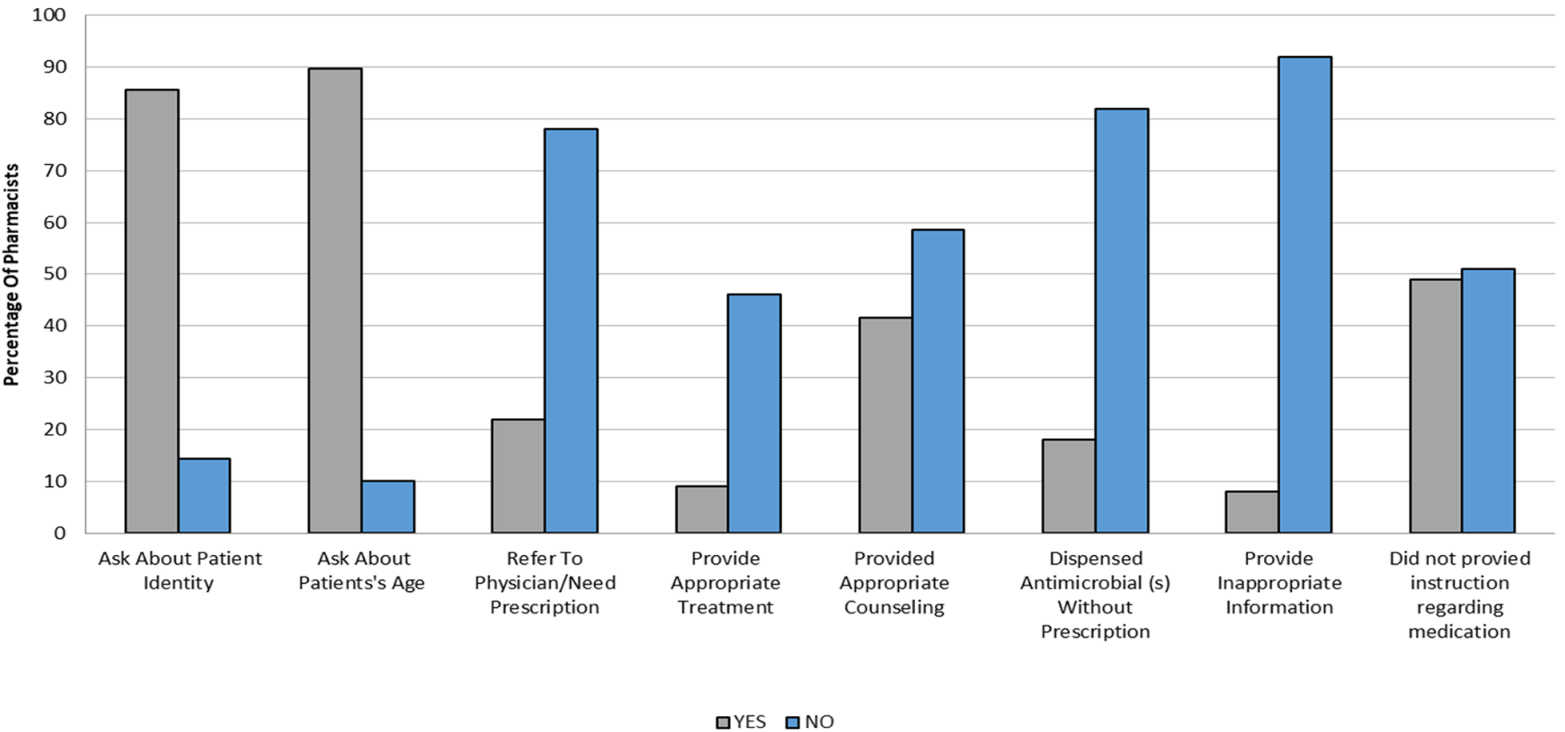
Percentage (%) of pharmacists who provide different types of patient assessment and treatment in all scenarios.

18% (*n* = 33) of pharmacists prescribed antimicrobials without a valid prescription, typically metronidazole (Figure 4). Regarding the pharmacists’ attitude toward customers, most pharmacists 57% (*n* = 104) showed a typical level of interest when interacting with simulated patients. However, 30% (*n* = 55) displayed a high level of interest, and 13% (*n* = 24) showed no interest at all.

The analysis revealed a significant difference in pharmacists’ practices based on the time period of the visit by the simulated patients (*P* = 0.001). Morning visits that occurred from 8 a.m. to 12 p.m. demonstrated the highest rate of appropriate practice at 73.3% compared to other times of the day. In contrast, afternoon visits from 12 to 4 p.m. had the lowest rate of appropriate practice at only 41% and the highest rate of poor practice at 59% across all time periods. Evening visits occurring from 4 to 8 p.m. and night visits from 8 p.m. to 12 a.m. showed moderate rates of appropriate pharmacist practice at around 50%. However, night visits had the highest rate of optimal practice at 8.1% compared to other time periods.

Othe factors including gender, nationality, type of pharmacy, and pharmacy location, did not show a statistically significant relationship with practices based on their *p*-values (0.088, 0.229, 0.598, and 0.322, respectively). Table 3 presents a cross-tabulation of the association between pharmacists’ characteristics and practices.

**Table 3:** Cross tabulation between pharmacist's characteristics and their practice.

Variables	Practice categories			*P* value
	Appropriate *n* (%)	Less appropriate *n* (%)	Poor *n* (%)	
Gender				0.088
Male	4 (2.3)	96 (54.9)	75 (42.9)	
Female	1 (14.3)	5 (71.4)	1 (14.3)	
Nationality				0.229
Saudi	3 (1.9)	86 (55.5)	66 (42.6)	
Non-Saudi	2 (7.4)	15 (55.6)	10 (37)	
Type of pharmacy				0.598
Independent	1 (1.4)	43 (58.9)	29 (39.7)	
Chain	4 (3.7)	58 (53.2)	47 (43.1)	
Pharmacy location				0.322
Close to Haram	4 (2.4)	94 (55.3)	72 (42.4)	
Away from Haram	1 (8.3)	7 (58.3)	4 (33.3)	
Period of visit				0.001
Morning (8 a.m.–12 p.m.)	0	22 (73.3)	8 (26.7)	
Afternoon (12 p.m.–4 p.m.)	0	16 (41)	23 (59)	
Evening (4 p.m.–8 p.m.)	0	24 (47.1)	27 (52.9)	
Night (8 p.m.–12 a.m.)	5 (8.1)	39 (62.9)	18 (29)	

## 4 Discussion

We believe this is the first study to fully evaluate current practices of community pharmacy professionals in Saudi Arabia in managing children with acute diarrhea. The findings suggest that a significant proportion of pharmacists in community pharmacies display suboptimal practices in managing pediatric diarrhea. Whilst this is similar to the findings from Ethiopia, which showed that community pharmacies do not provide adequate treatment for acute diarrhea in children (31), our findings suggest an urgent need to update the curriculum for student pharmacists in Saudi Arabia and beyond as well as instigate continual professional development (CPD) activities post qualification.

A noticeable finding though was the highest rate of optimal practice occurred during night visits (8 p.m.–12 a.m.) compared to other time periods despite the limited sample size. Several factors may explain this result. These included the availability of experienced pharmacists on night shifts and lower patient volumes enabling more dedicated counseling time per case (32, 33). However, this observation warrants validation through expanded studies across more locales and with larger samples to substantiate any associations between visit time and the quality of care of pediatric diarrhea, and we will be following this up in future research projects.

It was initially hypothesized by the research team that the first scenario would yield the highest percentage of adequate practices, given the clarity of the “red flag” for referral. However, this was not the case, and the highest percentage of poor practices was observed for this scenario indicating considerable deficiencies in the pharmacists’ clinical assessment skills and judgment regarding appropriate referral. Conversely, the third scenario exhibited the highest percentage of adequate practices. The context of this scenario was intentionally broader, with more acceptable management options.

Given the appreciable differences in intervention approaches between adults and children, it is essential to obtain all relevant patient information before suggesting possible approaches to manage pediatric diarrhea. Encouragingly, a significant proportion of surveyed pharmacists in our study were engaged in this behavior, reflecting professional practice. However, the lack of a designated space for confidential patient counseling in a number of pharmacies led to some pharmacists dispensing medications without adequately seeking additional information from the patient. Overall, only 62% of pharmacists recommended one or more medications for acute diarrhea treatment, with 20% of cases referred to physicians irrespective of the case characteristics. In addition, a small number of pharmacists abstained from making any recommendations, which is a concern. Unfortunately, this could reinforce the public perception that pharmacists are mainly engaged in medicine sales and may be incapable of assessing and treating minor conditions such as diarrhea (34). This is an issue that needs to be addressed especially with community pharmacists playing a key role in many countries during the COVID-19 pandemic including giving advice on preventative measures for infectious diseases and dealing with misinformation (17, 18, 35), and they need to maintain this role and respect into the future.

In all scenarios, the majority of dispensed medications included kaolin and pectin (34%), which is similar to the findings of Merghani et al. (2022) which reported a 43% recommendation for the same medication (16). Despite the World Health Organization's statement that routine use of probiotics for severe diarrhea in children is not recommended, some pharmacists still proposed their use as a potential therapeutic intervention. However, the effectiveness of such treatments in diarrhea management remains uncertain (1, 36, 37).

Another concern is that in the present study only 20% of pharmacists recommended ORS, and none suggested the use of zinc supplements. This was very different to the findings of Merghani et al. (2022), where 90% of community pharmacists in their study in Saudi Arabia recommended the use of ORS, with 59% also suggesting the inclusion of zinc supplements (16). Similarly, Ayele et al. (2018) reported that 33.3% of pharmacists in Gondar town, Ethiopia, recommended ORS with zinc. We are not sure of the reasons behind these appreciable differences in study findings and will be exploring this further in future studies in view of the concerns raised with the findings in our study.

We have seen in the study of Khojah that during the recent COVID-19 pandemic, 15.8% of pharmacies in Saudi Arabia dispensed an antibiotic without a prescription, higher than seen in the study of Alshreedy et al. (2020) at 12.1% of patients with pharyngitis, with a similar proportion in the study of Al-Tannir et al. (20, 24, 38). However, in the current study, the percentage was slightly higher at 18%, indicating a possible lax in the current monitoring of community pharmacies. Even in the absence of evidence to support its routine use, metronidazole was often suggested for managing pediatric diarrhea, except when it was specifically caused by a *Clostridioides difficile* infection (14). This misuse and overuse of antibiotics is a concern as this contributes to the emergence of AMR, which poses a significant threat to public health (39). Pharmacists play a vital role in ensuring the appropriate use of antibiotics (21, 40). They have a professional responsibility to provide treatment guidance following evidence-based guidelines and considering the specific needs of their patients. By vigilance and adhering to best practices, pharmacists can help combat rising rates of AMR by not dispensing antibiotics inappropriately without a prescription. This is in line with best practice as well as the regulations in Saudi Arabia and globally, with education of patients and pharmacists key to reducing inappropriate requests and/or dispensing of antibiotics without a prescription (21).

Regarding pharmacists’ readiness to assist patients, the study findings revealed a noticeable lack of engagement and enthusiasm among pharmacists working in rural, sparsely populated areas when fulfilling their professional responsibilities. This is also a concern going forward that needs to be addressed to continue to cement their relationship with patients. As a result, direct them towards optimal management of presenting infectious diseases and away from any requests for antibiotics thereby reducing AMR.

We believe this study possesses several strengths, including its methodological approach with simulated patients. The study was also conducted among community pharmacies serving a diverse population. The simulated patients utilized in this study were students enrolled in the Doctor of Pharmacy (PharmD) program who underwent comprehensive training in multiple study situations under the principal author's supervision. Consequently, helped to ensure realistic patients. However, we are aware this study had limitations. The data collection process was restricted to a single point in time thereby limiting the evaluation of temporal variations. Pharmacies that were untraceable or temporarily closed at the time of the client visit were excluded from the study. Given that pharmacy employee behavior may vary across different time periods, and each pharmacy was only visited once by a simulated patient, certain conclusions may lack conclusiveness due to the absence of public and patient feedback. Within the context of the pseudo-patient methods, it was noted that only a limited number of pharmacists provided instructions for each medication during the payment process. However, the absence of real-world scenarios in these methods may affect the precision of determining the exact proportion of pharmacists who neglect to provide instructions. We also could not fully assess the knowledge of pharmacists regarding the management of pediatric diarrhea using the simulated patient methodology. Alongside this, we may not always be able to fully determine the pharmacy model. The study was also only conducted in one Province for the reasons stated. Additionally, some of the observations and scoring relied on subjective assessments, which may have introduced bias. Despite these limitations, we believe our findings are robust providing direction for the future.

## 5 Conclusions

There were concerns regarding the management of pediatric diarrhea among community pharmacists in Madinah province, and potentially across Saudi Arabia, in our study. The concerns need addressing through modification of the curriculum with undergraduate pharmacists as well as additional education post qualification to improve the management of these priority patients. Alongside this, the unauthorized sale of antibiotics without a prescription is still an issue in Saudi Arabia exacerbating AMR that also needs to be addressed. In the first instance, it is recommended to strengthen the enforcement of regulations with appreciable fines for abuse alongside conducting additional educational programs among pharmacists and parents to enhance their clinical competency and increase their awareness of the appropriate management of pediatric diarrhea. By doing so, reduce the pressures on pharmacists to dispense antibiotics inappropriately thereby helping to reduce AMR.

## Data Availability

The original contributions presented in the study are included in the article/Supplementary Material, further inquiries can be directed to the corresponding authors.
